# Health-related stakeholders’ perceptions of clinical pharmacy services in Qatar

**DOI:** 10.1007/s11096-020-01114-0

**Published:** 2020-09-22

**Authors:** Tesnime Jebara, Scott Cunningham, Katie MacLure, Ahmed Awaisu, Abdulrouf Pallivalapila, Moza Al Hail, Derek Stewart

**Affiliations:** 1 School of Pharmacy and Life Sciences, Robert Gordon University, The Sir Ian Wood Building, Garthdee Road, Aberdeen, AB10 7GJ UK; 2 College of Pharmacy, QU Health, Qatar University, Doha, Qatar; 3 Pharmacy Department, Women’s Wellness and Research Center, Hamad Medical Corporation, Doha, Qatar

**Keywords:** Pharmacy practice, Clinical pharmacy, Service evaluation, Theory, Middle east, Qualitative study

## Abstract

*Background* In Qatar, the National Vision 2030 and the National Health Strategy 2018–2022 articulate the need to improve healthcare delivery by better utilisation of the skilled workforce. In this regard, pharmacy practice is rapidly advancing and several extended pharmacy services are now available in institutionalised settings. *Objective* This study aimed to determine health-related stakeholders’ perceptions of current clinical pharmacy services in Qatar, and the potential development and implementation of further patient-centred roles. *Setting* All major organisations and institutions relating to the practice, education, regulation, and governance of pharmacy in Qatar. *Method* Qualitative, face-to-face semi-structured interviews were conducted with individuals in key strategic positions of policy development and influence (i.e. health-related academic leaders, healthcare policy developers, directors of medicine/pharmacy/nursing, and patient safety leaders). Participants were recruited via a combination of purposeful and snowball sampling, until the point of data saturation was reached. The interview guide was grounded in the Consolidated Framework for Implementation Research domains of innovation characteristics, outer and inner setting, characteristics of individuals, and implementation process. The interviews were digitally recorded, transcribed and independently analysed by two researchers using the Framework approach. *Main outcome measure* Perceptions of stakeholders regarding current and potential for future clinical pharmacy services in Qatar. *Results* Thirty-seven interviews were conducted with stakeholders of policy influence in healthcare. The interviewees reported a variety of clinical pharmacy services available in Qatar, which they perceived as positively impacting patient care outcomes, pharmacists’ professional autonomy, and the healthcare system in general (innovation characteristics). However, they perceived that these services were mainly performed in hospitals and less in community pharmacy setting (inner setting) and were undervalued by patients and the public (outer setting). Expansion of pharmacists’ clinical activities was supported, with recognition of facilitators such as the skillset and training of pharmacists, potential time release due to automation and well-considered implementation processes (characteristics of individuals, inner setting, process). *Conclusion* Health-related stakeholders in Qatar have positive perceptions of current clinical pharmacy services and support the expansion of pharmacist’s roles. However, service development needs to consider the issues of patient and public awareness and initially target institutionalised healthcare settings.

## Impacts on practice

- In Qatar, pharmacy practice is rapidly advancing and several extended clinical pharmacy services are now available in institutionalised healthcare settings.
- However, limited evidence is available to explore how key health-related stakeholders perceive these services including the potential development and implementation of new patient-centred pharmacist’s roles.
- Findings of this research could facilitate cognitive pharmaceutical service development and better provision of patient-centered care, which could be transferrable to other countries, especially from the context of the Middle East.

## Introduction

The evolution from product focused pharmacy services to more patient-centred pharmacy services was articulated in World Health Organization (WHO) policy documents advocating better use of pharmacists’ skills to improve health care [1–3]. Similarly, the International Pharmaceutical Federation (the global body representing pharmacy, pharmaceutical sciences and pharmaceutical education) advocates the systematic development and implementation of more clinical patient-centred and outcomes-oriented services [4, 5].

Several countries have well-developed clinical pharmacy services which are part of routine practice. For instance, in the United Kingdom (UK), clinical pharmacy services have evolved from rarely providing therapeutic drug monitoring and medication history-taking to the introduction of supplementary prescribing in 2003 and independent prescribing in 2006 [6, 7]. The majority of literature evaluating pharmacist prescribers’ practice demonstrates safety, the achievement of clinical outcomes comparable to physicians [8, 9] and acceptability by a range of key health stakeholders [10, 11]. In 2015, the UK launched an ambitious plan aiming to embed clinical pharmacists within general medical practices [12, 13]. An independent evaluation based on pilot data from England confirms the significant contribution to patient safety, supporting long-term condition management, and improving medication knowledge within the team [14]. Similar positive results have been reported in Scotland, where pharmacists are now practising within one-third of general medical practices [15].

Over the past decade, Qatar has taken steps to transform its healthcare system. The Qatar National Vision 2030 aims to “transform Qatar into an advanced country by 2030, capable of sustaining its own development and providing for a high standard of living for all of its people for generations to come” by “balancing the accomplishments that achieve economic growth with the human and natural resources” [16]. To realise the ambitions of the National Vision for a healthy population, the National Health Strategy was launched in 2011 [17]. Six projects were initiated, one of which aimed to strengthen the role of pharmacists in supporting patients, as well as improving the quality of healthcare system by making it more accessible and less costly. Recently, the Ministry of Public Health reinforced the shift in care provision from secondary and tertiary care to primary care in an updated National Health Strategy 2018–2022, “Our Health, Our Future: Improved health for Qatar’s population, meeting the needs of existing and future generations” [18].

**Fig. 1:**
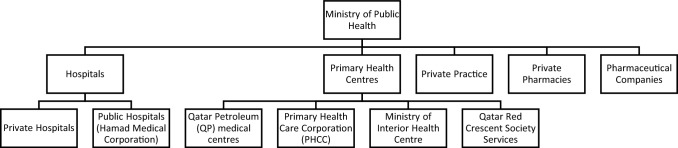
Healthcare system in Qatar [20]

Healthcare in Qatar is provided through government-funded and private-provided secondary and tertiary institutions, government-funded primary healthcare centres and private sector centres (ambulatory care), and community pharmacies (Fig. 1) [19].

Hamad Medical Corporation (HMC) is the largest provider of healthcare in Qatar, and is regarded as one of the leading hospital services providers in the Middle East region. HMC manages 12 hospitals—nine specialist hospitals and three community hospitals—as well as the National Ambulance Service and home and residential care services [21]. In 2016, HMC became the first healthcare system across the globe to have all its hospitals accredited by Joint Commission International (JCI) under the Academic Medical Center accreditation program [21].

Pharmacy practice in Qatar has advanced in recent years due to multiple factors including the establishment of the first national college of pharmacy in 2007 [22]. As at 2013, the number of pharmacists registered in Qatar was 1023 in the public and 991 in the private sectors giving an estimated 1.01 pharmacists per 1000 population [23, 24]. There is some evidence that pharmacy services are evolving toward clinical, patient-centred roles [25]. HMC has implemented a pharmacist-led anticoagulation clinic, with initial positive findings for clinical outcomes of time in therapeutic range (TTR), percentage of visits within therapeutic range and reduced percentage of visits with extreme subtherapeutic international normalized ratio (INR) [22, 26]. Therefore, pharmacy practice is rapidly advancing and several extended pharmacy services are now available in institutionalised settings in Qatar. There, however, remains potential for expanding further clinical pharmacy services to all sectors of practice. Obtaining the perceptions of key decision-makers (stakeholders) would facilitate plans for expansion.

### Aim of the study

This study aimed to determine health-related stakeholders’ perceptions of current clinical pharmacy services in Qatar, and the potential development and implementation of further patient-centred roles.

### Ethics approval

Ethical approval was granted by Robert Gordon University School of Pharmacy and Life Sciences Research Ethics Committee (Approval reference S64), Ministry of Public Health Ethics Committee, Hamad Medical Corporation Medical Research Committee (Approval reference MRC0449/2017), and Qatar University Institutional Review Board (Approval reference QU-IRB 772-E/17).

## Methods

### Study design

This study utilised a qualitative approach of semi-structured interviews with key stakeholders. A qualitative methodology was selected to generate in-depth, rich data allowing detailed description and understanding of perceptions around clinical pharmacy practice in Qatar [27].

### Setting

Data generation took place across all major organisations and institutions relating to the practice, education, regulation, and governance of pharmacy practice in Qatar. These comprised of the Ministry of Public Health; primary, secondary and tertiary healthcare institutions; community pharmacies; and all healthcare academic institutions providing pharmacy, medical, nursing, and pharmacy technician education programmes.

### Inclusion and exclusion criteria

The intention was to generate data representing all key stakeholder groups, with policy influence relating to the development, implementation and evaluation of clinical models of care. The groups targeted were individuals in key strategic positions of policy influence (i.e. health-related academic leaders, healthcare policy developers, directors of medicine/pharmacy/nursing, and patient safety leaders). Members of the research team based in Qatar, who might have satisfied the above criteria, were excluded from the study.

### Sampling and sampling approach

The sampling frame included all individuals meeting the inclusion and exclusion criteria. The names and contact details of all potential participants were collated by the research team members using their professional networks. Purposive sampling was employed to capture those individuals who were most likely to contribute to the achievement of the research objectives. Snowball sampling was also utilised to ensure that no key individuals had been omitted by asking each interviewee to recommend others that they thought were important to include. Sample size was determined to achieve data saturation according to the steps outlined by Francis et al. [28] Five members from each professional group were interviewed as the initial analysis sample. This sample was believed to represent adequate diversity based on the participants’ professions. Afterwards, one additional interview was performed for each professional group before the stopping criterion was tested. Responses were coded and analysed independently by two research team members in order to confirm that data saturation was achieved.

### Development of interview guide

The semi-structured interview guide was constructed based on a review of the published literature on pharmacy practice and grounded in the Consolidated Framework for Implementation Research (CFIR) [29]. This theoretical framework was used to ensure that all relevant implementation factors that can determine the success or failure of any innovation are deliberated. The guide was developed and mapped against the five main domains of CFIR: innovation characteristics (of the intervention being implemented into a particular organisation), outer setting (i.e. economical, political, and social context within which an organisation resides), inner setting (i.e. structural, political, and cultural context through which implementation process will proceed), characteristics of individuals (who will enact the intervention), and process (of implementation) [29]. The interview guide included questions related to participants’ awareness of current clinical roles performed by pharmacists in Qatar, their views on the efficiency of these services and any potential roles pharmacists in Qatar can perform in the future. The guide was reviewed for dependability, which is the degree to which different researchers are able to produce the same data [30], by members of the research team prior to piloting with five academic and practice-based stakeholders. This pilot process did not lead to any major changes to the guide. Pilot interviews were not included in the dataset.

### Data generation

All potential participants were e-mailed the information leaflet, with a reminder sent 2 weeks later if no response had been received. The interviews were conducted from April to August 2017 by one of the research team members (TJ) who was trained in conducting qualitative research. Written, informed consent was obtained from all research participants before commencing. Interviews, of approximately 45–60 min, were audio-recorded and later transcribed by TJ and reviewed for accuracy by DS. All interviewees were offered the opportunity to review the transcripts to promote credibility and dependability [30].

### Data analysis

Thematic analysis was conducted using the framework approach following the steps outlined by Ritchie and Spencer [31]. All interviews were input into NVivo^®^ software then initially coded by TJ based on the different constructs of the CFIR before being examined for further sub-themes within each construct. Afterwards, the emerging themes were reviewed against the original data then indexed and refined. The analysis was reviewed by another team member to ensure credibility, with any disagreements resolved through discussion.

## Results

### Stakeholders recruitment

Out of 74 stakeholders invited, 37 interviews were conducted to reach data saturation in each stakeholder group giving representation of all practice settings (Table 1).

**Table 1:** Characteristics of included stakeholders according to practice setting

Stakeholders’ category	Setting: number of participants*
Academic leaders	Medicine: 2 Pharmacy and Pharmacy technician: 5 Nursing: 2
Healthcare policy developers	Primary care/Community: 1 Secondary care: 1 Tertiary care: 2 Corporate/Ministry: 2
Medical practice leaders	Primary care/Community: 1 Secondary care: 3 Tertiary care: 5 Corporate/Ministry: 1
Pharmacy practice leaders	Primary care/Community: 3 Secondary care: 1 Tertiary care: 3 Corporate/Ministry: 1
Nursing practice leaders	Secondary care: 1 Tertiary care: 5 Corporate/Ministry: 3
Patient safety advocates	Primary care/Community: 1 Secondary care: 1 Tertiary care: 4

*Some held multiple roles within their organisation

### Key themes that emerged from the interviews

Themes which emerged during the interviews were mapped to the different CFIR domains. The major themes frequently highlighted by interviewees are described in more details below while the rest are summarised in Table 2. There were no marked differences in responses across the professional groupings.

**Table 2:** A summary of the CFIR domains and constructs relating to views and perspectives on pharmacy practice in Qatar

CFIR domain	CFIR constructs	Key themes		Illustrative quotes
**Innovation characteristics** of the intervention being implemented into a particular organisation	**Evidence strength and quality** Perceptions on quality and validity of evidence supporting the innovation	The expansion of the numbers of clinical pharmacist within HMC was considered to be an indication of effectiveness, efficiency and acceptability		*I don’t really have the statistics but the number of clinical pharmacists now in Hamad [Hamad General Hospital] is really big. We started only with 10. Today maybe we are talking about 60*–*70 pharmacists. So why did we have this expansion if the role is not really important?* Academic Leader 3
**Outer setting** i.e. economical, political, and social context within which an organisation resides	**Needs and resources** Extent to which patient needs are known and prioritised	There was a feeling that the general public was not aware of the skillset and roles of pharmacists and that this could act as a barrier to expanding scope of practice		*Patients are barriers because they don’t really value the role of the pharmacist… Anytime you speak about pharmacist, shop keeper image pops to their mind*. Academic Leader 1
**Inner setting** i.e. structural, political, and cultural context through which implementation process will proceed	**Networks and Communications** Nature and quality of networks and communications within the organisation	There were mixed views on the degree of integration of the pharmacist within the multidisciplinary team and therefore the communication networks in operation		*I have really good relationship here with my pharmacist as well. We have dialogue with regard to if there is any issue with nurses and administration for instances as well.* Nursing Practice Leader 3 *In the psychiatric hospital, the pharmacist was always in a little back room. And not on the floor at all*. Academic Leader 8
		Trust, relationships and communication were thought key to the anticoagulation clinic		*From the beginning we had very strong and very positive relationship with the medical directors…They are trusting me a lot. Building the trust and having good relationships with the team will facilitate everything*. Pharmacy Practice Leader 6
	**Culture** Norms, values and basic assumptions of the organisation	Despite the positive developments in clinical practice in HMC, there was still the view that pharmacists were generally undervalued		*I suspect that there is a traditional view about the dispensing type pharmacist rather than that understanding of them having the kind of clinical background knowledge as well*. Nursing Practice Leader 1
		One factor thought contributing to this lack of awareness and lack of value was the large number of expatriated health professionals		*We have physicians from very different backgrounds and parts of the world and all the different cultural heritages and there are sometimes adjustments needed to understand that other professional groups are just as valuable as their professional group is*. Medical Practice Leader 1
	**Implementation climate** Absorptive capacity for change	Tension for change	Almost all acknowledged that while clinical practice had developed, there was potential for further developments	*Definitely there is a lot of room for improvement. There has been quite a lot of changes to the pharmacist role but there is a lot of space for further improvement*. Academic Leader 1
		Relative priority	Developments in clinical practice were felt to align to the Qatar National Vision hence were considered beneficial	*…It will relieve the physician time a little bit… I think this is a very important part to be considered in the priorities of the national health over here*. Academic Leader 6
	**Readiness for implementation** Tangible and immediate indicators of organisational commitment to implementation	Leadership engagement	The support of the pharmacy leaders was considered a main influential factor in clinical development	*I think the Director of Pharmacy, she is really a pioneer in introducing this programme [clinical pharmacy] into Women’s Hospital. This started I think seven years ago and now we have very good clinical pharmacists who are eager to work and to do further responsibilities.* Medical Practice Leader 3
		Available resources	There was a perception that the current number of skilled clinical pharmacists was a barrier to further service development	*You may have enough [pharmacists] but you need specific skills, you need people with clinical practice, competencies and clinical background. Qatar lacks the number of clinically qualified and skilled professionals.* Pharmacy Practice Leader 6
			This was noted to be a particular issue during times of staff absences	*If you have only one pharmacist covering the service, it gets difficult sometimes when they need to be on leave for conference or vacation.* Academic Leader 6
**Characteristics of individuals** Involved in enacting the intervention	**Knowledge and beliefs about the innovation** Individuals’ attitudes and value placed on innovation	There was general agreement that, given their education and training, pharmacists should be maximising their input to patient care		*When I look at the education of pharmacists, I think they should be working to full scope… I think by maximising their scope, it is better for the system and it is better for the patients.* Academic Leader 9
	**Self-efficacy** Individual beliefs in their own capabilities	There was recognition that while some pharmacists were motivated to develop their clinical practice, there were many likely to be less so		*Pharmacy like any other profession, you will find the group of people who are really motivated to do more work, love the profession, would like to serve more …and other pharmacists who are still [not].* Academic Leader 3
		Notably those involved in establishing the anticoagulation clinic were highly committed despite their existing workload		*The people I had on the team were very committed and enthusiastic to the project despite being already overwhelmed with their original work. They were working very hard and they were very proud.* Pharmacy Practice Leader 6
	**Other personal attributes** Broad construct to include other traits such as motivation, value, competence, capacity and learning style	The personal development of the pharmacists in Qatar was appreciated and thought related to their extensive training, and rigid recruitment and licensing standards		*I think they improved a lot from maybe 17*–*18* *years ago. There is a lot of improvements in pharmacy practice and we appreciate that there is this change and improvement.* Healthcare Policy Leader 6 *We have a very tight process for recruiting pharmacists so we are looking for quality first. So we have very good quality pharmacists with us.* Pharmacy Practice Leader 2
		There was, however, recognition that practice in community pharmacy centred on dispensing		*The role that is played by the community pharmacists in Qatar is still the traditional role in which they just sell medications and maybe provide some recommendations or counselling from time to time.* Academic Leader 3
**Process** of implementation	**Planning** Degree to which a scheme or method for implementation are developed in advance	When developing and implementing clinical pharmacy services, there were several key factors for successful implementation such as the need for communication with other health professionals		*From the beginning, before we started anything, we communicated in a very nice way with the physicians… So none of the physicians felt threatened… We were very scientific, very friendly with them.* Pharmacy Practice Leader 6
		Establishing clear guidelines during the development of the anticoagulation clinic and continuous monitoring after implementation were cited as being important		*We developed the guidelines for all of the anticoagulants and antiplatelets and we decided on and approved the workflow as well… And then we started and we continued monitoring the work, take feedback from physicians and from patients to improve the process.* Pharmacy Practice Leader 6
	**Engaging (Key stakeholders)** Attracting and involving appropriate individuals in implementation and use of innovation	The need to engage the right key stakeholders as part of implementation was highlighted		*And the consultant here was very happy about this idea and he supported it especially since he came from Canada and he knew how the system was running and he supported this. Otherwise it wouldn’t have worked.* Patient Safety Advocate 4

Interviewees noted a significant evolution in clinical pharmacy services in Qatar, illustrated by the continuous implementation of new services such as the pharmacist-run anticoagulation clinic with advantages to patients and pharmacists themselves (CFIR domain: innovation characteristics). These developments were perceived as being carefully planned with clear communications, guidelines, and monitoring processes (CFIR domain: process). The interviewees perceived that these services were mainly performed in hospitals and less in community pharmacy setting (CFIR domain: inner setting). However, a lack of patient and public awareness regarding clinical pharmacy activities was also highlighted (CFIR domain: outer setting). In addition, some stakeholders reported that the majority of clinical pharmacy services were performed exclusively in hospitals with community pharmacy setting mainly focused on traditional pharmacist’s product-oriented role of drugs dispensing. Nevertheless, the interviewees highlighted the potential for further development, aligned to the Qatar National Vision and supported by healthcare leaders (CFIR domain: inner setting). They also commented on the quality of the education and training of pharmacists and the strict licensing requirements to practice in Qatar (CFIR domain: characteristics of individuals).

Given that most discussion (based on counts and depth of discussion) centred on specific CFIR domains and constructs, these are considered in more detail. These were: innovation source (perception of key stakeholders about whether the intervention is externally or internally developed); relative advantage (stakeholders’ perception of the advantage of implementing the intervention versus an alternative solution); design quality and packaging (perceived excellence in how the intervention is bundled, presented, and assembled); structural characteristics (the social architecture, age, maturity, and size of an organization); and compatibility (the degree of tangible fit between meaning and values attached to the intervention by involved individuals, how those align with individuals’ own norms, values, and perceived risks and needs, and how the intervention fits with existing workflows and systems).

#### Innovation characteristics

In terms of the innovation of clinical pharmacy practice, there was recognition that this was developing at pace, particularly within HMC. There was description of the clinical pharmacy service in general and exemplified through specialist activities such as the pharmacist-led anticoagulation clinic.

1. Innovation source

There was recognition that the anticoagulation service had developed significantly in recent years. This was expressed by participants from all stakeholder groups.

*The clinical pharmacists’ role has developed quite significantly in recent years like, for example, in [Name] Hospital there is the anticoagulation clinic that is run by pharmacists.*

*Academic Leader 1*

The clinical service had extended to allow dose adjustment.

*We call it the anticoagulation clinic where the clinical pharmacists are on the front line, meeting the patients…checking the INR and then adjusting the dose, so they make the decision.*

*Medical Practice Leader 5*

There was recognition of other HMC clinical services.

*Also in clozapine clinic in mental health, there is a direct contact with the patient for monitoring the side effects and recording if require changing the dose…*

*Pharmacy Practice Leader 4*

2. Relative advantage

Clinical practice was perceived to bring advantages including professional autonomy, which was considered important to further developments.

*… it will give you more confidence in what you are doing, your clinical skills, would help increase the trust that is given to the pharmacists and maybe help them gain more roles in the future.*

*Academic Leader 6*

There were also examples of positive feedback from patients at HMC.

*Generally our feedback with like warfarin clinics and such that we have is very positive… patients don’t wait for months to get an appointment.*

*Healthcare Policy Leader 2*

This clinical service was also reported to reduce pressure on physicians.

*The most important advantage I see from the side of physicians is that it will free their time to do more skilled work.*

*Academic Leader 6*

There was also the view that patient health outcomes were improving.

*We can see definitely that the anticoagulation outcomes have been better with those monitored by pharmacists.*

*Academic Leader 6*

3. Design quality and packaging

Mandatory continuing professional development for pharmacists was considered a positive development, contributing to the quality of services.

*I think they are efficient. They keep continuous education so they have weekly sessions so they are updating themselves.*

*Healthcare Policy Leader 1*

Furthermore, those involved in the delivery of new and specialised services had to undertake further education and training.

*It is a condensed course that focuses on anticoagulation, how to dose, what are the guidelines available and the pathophysiology.*

*Pharmacy Practice Leader 3*

While these developments were in HMC settings, it was highlighted that they could also be in others such as in primary healthcare centres.

*They don’t have it in primary healthcare, the warfarin clinic. But if the volume of patients increase, it can be in primary healthcare, why not?*

*Medical Practice Leader 5*

#### Inner setting

1. Structural characteristics

Many participants highlighted that while the public sector was highly regulated with focus on quality of care, the private sector was profit-oriented.

*The public sector I think it is more structured because they are accredited… but in private, nothing. They don’t have the policies, they don’t have guidelines, they are more or less looking for money.*

*Healthcare Policy Leader 3*

There were contradictory views around the influence and power of the medical profession around these developments in clinical pharmacy practice.

*It is still completely physician driven. There is still a hierarchy.*

*Academic Leader 8*

*In the cancer centre they have a very intimate collaboration with them [physicians] and communication and they really are valued [as an] equivalent member of the team. There is no hierarchy.*

*Medical Practice Leader 1*

One overwhelming theme was that clinical pharmacy practice varied greatly with setting. There was recognition that HMC was much further advanced than other settings.

*As for practice, hospitals are better than community since community is not well developed because it lacks assessment and clinical practice.*

*Pharmacy Practice Leader 6*

*In primary healthcare centres, they are developing now, they are in a better position if you compare them to the community pharmacists. But I have to say that still they need to work more on themselves.*

*Academic Leader 3*

2. Implementation climate
  - Compatibility

There was some appreciation that the clinical pharmacy services in HMC could be benchmarked to practice in western countries.

*I left the UK thinking that I had a very good pharmacy service. I have come to Qatar and there is a better pharmacy service here in my view.*

*Healthcare Policy Leader 5*

The level of technology and automation available within HMC was considered a positive facilitator of development.

*The level of automation that goes into packages like Cerner is clearly already demonstrating that pharmacists can take on a more assertive role on behalf of the patients to protect their safety…*

*Healthcare Policy Leader 4*

While the clinical anticoagulation clinic was well-established, it was noted that this took time, with acceleration once physicians had evidence of positive outcomes.

*At the beginning, when they don’t have much experience with it, they may be a little bit suspicious… but then after a while, after they saw our abilities and how really the patients have been stable and monitored appropriately, I think they got more and more confident.*

*Academic Leader 6*

## Discussion

Key stakeholders in Qatar perceived clinical pharmacy services as having a positive impact on patients’ outcomes, pharmacists’ professional autonomy, and the overall healthcare system. However, interviewees did highlight differences in the extent of the implementation with tertiary and secondary public care (mainly HMC) being the most advanced. Interviewees perceived a lack of awareness of the extent of knowledge, training and practice of pharmacists in Qatar and that pharmacists were undervalued by patients and the public. There was potential to advance clinical pharmacy services, with recognition of facilitators such as the skillset and training of pharmacists, potential time release due to automation and well-considered implementation processes.

Pharmacists’ contribution to the healthcare system, the multidisciplinary team and thus patient care, aligns to the aspirations of the Qatar National Vision [16], its associated health strategies [17, 18] and international efforts for advancing patient-centred pharmacy practice [1–5].

Several aspects of healthcare setting emerged in this study as being key to the development and perceived success of patient-centred clinical pharmacy services, relating to CFIR domains of innovation characteristics, inner setting, outer setting, characteristics of individuals and process. This is not unique to Qatar, with the WHO and the International Pharmaceutical Federation highlighting that services are most likely to advance in secondary and tertiary care settings [3]. There are several likely contributory factors, including close multidisciplinary collaboration stimulating mutual trust and respect, ease of access to patient data and other key resources, peer support, leadership and aspects of governance including role definition, education, training and continuing professional development. Tonna et al. [32] noted the importance of these factors in defining a guide to planning the implementation of pharmacist prescribing, an example of a clinical pharmacy service, in UK hospital practice.

Barriers for clinical pharmacy implementation in primary care such as lack of awareness of pharmacists’ skillset and their potential clinical roles were also reported in a qualitative study in Canada [33]. The study highlighted that healthcare professionals were generally unaware of clinical pharmacy roles, which subsequently affected implementation. Similar findings were also reported in relation to the community setting. For instance, a systematic review of factors influencing national implementation of innovations within community pharmacy described three key influences which are: pharmacy staff engagement with the proposed services, operationalisation of the innovations (including design, complexity and resources needed), and pharmacy staff relationships with patients and other healthcare professionals [34]. Thus, there is a need to improve the provision of clinical pharmacy services in primary and community settings which should take into consideration theoretical frameworks, such as the CFIR, which highlight the importance of thorough development and the need for piloting and continuous evaluation in order to ensure the successful implementation of any future clinical pharmacy roles [29].

Pharmacist role expansion was facilitated mainly by the perceived level of education, training, and motivation that pharmacists possess which can further enable them to provide more clinical services in the future (CFIR domain: characteristics of individuals). This could be attributed to the increased focus on developing pharmacists’ skills in Qatar highlighted in the National Vision and National Health Strategy and evidenced by the level of pharmacy education offered in the state, the rigorous accreditation, licensing and continuous education training pharmacists in Qatar must undergo [22]. Similar facilitators related to pharmacists’ characteristics, mainly their motivation and knowledge, were also reported in a qualitative study with health-system pharmacists and managers who experienced the implementation of clinical pharmacy services in Brazil, highlighting the importance of having a skilled workforce for the successful implementation of such roles [35].

Implementing more advanced clinical pharmacy services, such as the pharmacist-managed anticoagulation clinic, was noted to be met with some resistance that was quickly overcome post-implementation. Zaidan et al. [36] also reported that physicians were more likely to accept new roles performed by pharmacists if they had exposure to these contemporary pharmacy services. These findings were also reported in the UK when pharmacists advocated for a prescribing role in relation to physicians’ concerns around liability and pharmacists’ abilities to perform such a role [11]. These findings highlight the importance of the lived experiences and the need for thorough planning and involvement of key stakeholders which also constitutes a key domain of CFIR thus highlighting the need to use a theoretical framework in the early development of any new innovation.

Despite these advances in clinical pharmacy services in Qatar, interviewees noted that the lack of public understanding of pharmacists’ knowledge, training and services in general is a major barrier to further role development especially within community pharmacy setting where pharmacists are considered to be drug dispensers (CFIR domain: outer setting). Low public or patient awareness of extended pharmacy services in the community pharmacy setting was also reported to be a common finding in the UK according to a recent systematic review of the current research literature focusing on perspectives on existing and future community pharmacy services [37]. This was often related to lack of exposure or utilisation of community pharmacy services. Thus, there is a great need to improve the public’s awareness of the real extent of pharmacists’ knowledge and skillset as well as the different roles they perform in order to allow them to expand their clinical services. This was considered especially important in Qatar where pharmacists are advocating for more clinical roles including prescribing. Recent studies have shown that key health stakeholders supported implementation of pharmacist prescribing in Qatar if additional training was provided and more efforts were made to increase awareness of the different services pharmacists provide in order to overcome potential resistance [20, 38].

This was a theory-driven, qualitative study generating in-depth, rich data of stakeholders’ perceptions. Steps were taken to enhance the trustworthiness (credibility and dependability) of the research data and findings, including adopting appropriate, well-recognised research methods, encouraging interviewees to be frank and talk freely and use of different researchers to independently analyse data. The key limitations are that the research outcomes are perceptions of stakeholders, rather than objective measures of service outcomes and that the findings may not be transferable beyond Qatar.

Further research should focus on the systematic development, implementation and evaluation of any future clinical pharmacy service development in Qatar.

## Conclusion

Health-related stakeholders in Qatar have positive perceptions of current clinical pharmacy services and support the expansion of pharmacist’s roles. However, service development needs to consider the issues of patient and public awareness and initially target hospital settings.
